# Molecular Mechanism of Caspase‐8–Dependent Interleukin‐18 Activation in Pancreatic Cancer Cells Induced by 5‐Fluorouracil and Nutrient Starvation

**DOI:** 10.1111/gtc.70111

**Published:** 2026-04-06

**Authors:** Hiroki Kamino, Yuko Nariai, Takeshi Urano

**Affiliations:** ^1^ Center for Vaccines and Therapeutic Antibodies for Emerging Infectious Diseases Shimane University Izumo Shimane Japan; ^2^ mAbProtein Co. Ltd Izumo Shimane Japan

**Keywords:** 5‐fluorouracil, caspase‐8, IL‐18, nutrient starvation, pancreatic cancer, pyroptosis

## Abstract

Interleukin‐18 (IL‐18) is a pro‐inflammatory cytokine, and higher IL‐18 expression in pancreatic tumors is associated with poor prognosis. Although 5‐fluorouracil (5‐FU) has been reported to induce the release of bioactive (mature/cleaved) IL‐18 from the pancreatic cancer cell line Capan‐2, the underlying mechanism remains unclear. Here, we investigated IL‐18 activation in pancreatic cancer cells after 5‐FU treatment under low‐nutrient conditions that mimic key features of the tumor microenvironment, using a monoclonal antibody we generated that specifically recognizes cleaved, active IL‐18. We detected the release of active IL‐18 from both Capan‐2 and MIA PaCa‐2 cells after 5‐FU treatment. Analysis of separated attached and detached cell fractions showed that IL‐18 cleavage occurred predominantly in detached cells. We also clarified that caspase‐8—but not caspase‐1/4—was activated in detached cells and was required for IL‐18 and GSDMD cleavage that is a hallmark of pyroptosis. Surprisingly, detached cells from only nutrient starvation showed the same phenomenon, and 5‐FU contributed to increased pyroptotic cells. On the other hand, the release of active IL‐18 was not observed with gemcitabine. These findings suggest that a low‐nutrient tumor microenvironment and 5‐FU therapy can promote caspase‐8–dependent pyroptotic cell death with IL‐18 activation, potentially contributing to chronic inflammation in pancreatic tumors.

## Graphical abstract

Nutrient starvation triggers pyroptosis with cleavage of pro‐IL‐18 and GSDMD in pancreatic cancer cells, and treatment of 5‐FU enhances this process. As a result, active IL‐18 is released and leads to inflammation.

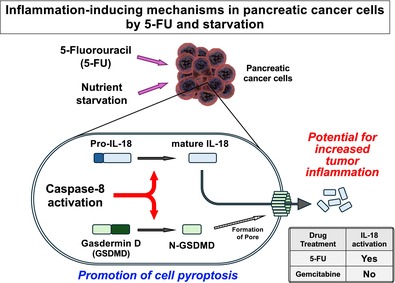

**Abbreviations**
5‐FU: 5‐fluorouracil
ASC: apoptosis‐associated speck‐like protein containing a CARD
GSDMD: gasdermin D
IL: interleukin
LDH: lactate dehydrogenase
MDSC: myeloid‐derived suppressor cell
PDAC: pancreatic ductal adenocarcinoma
PRR: pattern recognition receptor

## 1 Introduction

Interleukin‐18 (IL‐18) is an inflammatory cytokine of the IL‐1 family, closely related to IL‐1β (Dinarello 2009; Garlanda et al. 2013). In many cell types, IL‐18 is constitutively expressed as an inactive precursor (pro‐IL‐18), not only in immune cells (e.g., monocytes, macrophages, and dendritic cells) but also in non‐immune cells such as endothelial cells, intestinal epithelial cells, and keratinocytes (Nakanishi et al. 2001). The activation and extracellular release of mature IL‐18 are influenced by a phenomenon called “Pyroptosis” (Vande Walle and Lamkanfi 2016; Man et al. 2017). Pyroptosis is a type of inflammatory programmed cell death and defined as following steps. At first, pyroptosis is activated by signals such as bacteria, viruses, or damage‐induced inflammatory mediators (DAMPs), then cytosolic multiprotein complexes termed inflammasome is formed (Man and Kanneganti 2015). Subsequently, the activation of caspase occurred and cleaves the inactive inflammatory cytokine such as pro‐IL‐1β or pro‐IL‐18 into the mature, active form of IL‐1β or IL‐18 (Yu et al. 2021; Hayward et al. 2018). In parallel, inflammatory caspases cleave gasdermin D (GSDMD), liberating its N‐terminal fragment, which forms membrane pores and facilitating extracellular release of mature IL‐18 (Shi et al. 2015; Xia et al. 2021). During the pyroptotic process, caspase‐1 is involved in the canonical pathway, while caspase‐4/5/11 are involved in the non‐canonical pathway (Yu et al. 2021). Recently, caspase‐8 has also been suggested to be involved in the pyroptosis pathway (Malireddi et al. 2019). Ultimately, the cell ruptures and dies, which is defined as pyroptotic cell death. Released active IL‐18 through this process binds to the IL‐18 receptor and activates NF‐κB and MAPK signaling, inducing downstream inflammatory mediators and amplifying inflammatory responses (Adachi et al. 1998; Volin and Koch 2011).

Pancreatic ductal adenocarcinoma (PDAC) remains one of the most lethal malignancies worldwide (Pook and Pauklin 2021). Chronic pancreatitis is a known risk factor for pancreatic cancer, highlighting a strong link between inflammation and tumorigenesis (Guerra et al. 2007; Kolodecik et al. 2014; Shi and Xue 2019). Increased plasma IL‐18 has been reported in patients with pancreatitis and pancreatic cancer compared with healthy volunteers (Schneider et al. 2006; Guo et al. 2016), and IL‐18 elevation has also been observed in other cancers. However, the cellular source and mechanism responsible for IL‐18 activation in tumors have not been fully elucidated. In this context, Carbone and colleagues reported that 5‐fluorouracil (5‐FU) induces production of bioactive IL‐18 in pancreatic cancer Capan‐2 cells (Carbone et al. 2005). We therefore hypothesized that 5‐FU induces pyroptosis in pancreatic cancer cells, leading to release of mature IL‐18. In addition, we examined using low‐nutrient culture conditions designed to model nutrient restriction in the tumor microenvironment (Vaupel et al. 1989; Brown and Giaccia 1998).

Here, we demonstrated that activation of IL‐18 and cleavage of GSDMD in pancreatic cancer MIA PaCa‐2 cells were observed more strongly in detached cells than in attached cells after 5‐FU treatment under low‐nutrient culture condition. This phenomenon was also induced by nutrient starvation. Intriguingly, caspase‐8 activation rather than caspase‐1/4 activation was detected, which may differ from canonical or non‐canonical inflammasome‐mediated pyroptosis. We further explored whether this mechanism extends to cancer cell types beyond pancreatic cancer. Collectively, our findings suggest that elevated IL‐18 observed in cancer patients may reflect pyroptosis triggered by nutrient‐restricted tumor microenvironments and enhanced by anticancer 5‐FU therapy.

## 2 Results

### 2.1 IL‐18 Is Activated in Human Pancreatic Cancer Cells by 5‐FU Under Low‐Nutrient Culture Conditions

We recently reported novel anti‐human IL‐18 monoclonal antibodies (Nariai et al. 2019). The mAb 11‐4.1 recognizes both full‐length IL‐18 (24 kDa) and cleaved IL‐18 (18 kDa), whereas mAb 9‐10.2 specifically recognizes the N‐terminal neoepitope generated upon inflammatory caspase‐mediated cleavage of IL‐18 (e.g., caspase‐1/4) (Nariai et al. 2019). Because Carbone et al. showed that Capan‐2 cells secrete bioactive IL‐18 after 5‐FU treatment (Carbone et al. 2005), we tested whether we could detect cleaved IL‐18 using our antibodies. To better reflect the low‐nutrient tumor microenvironment (Vaupel et al. 1989), we performed experiments in low‐nutrient culture medium.

**FIGURE 1:**
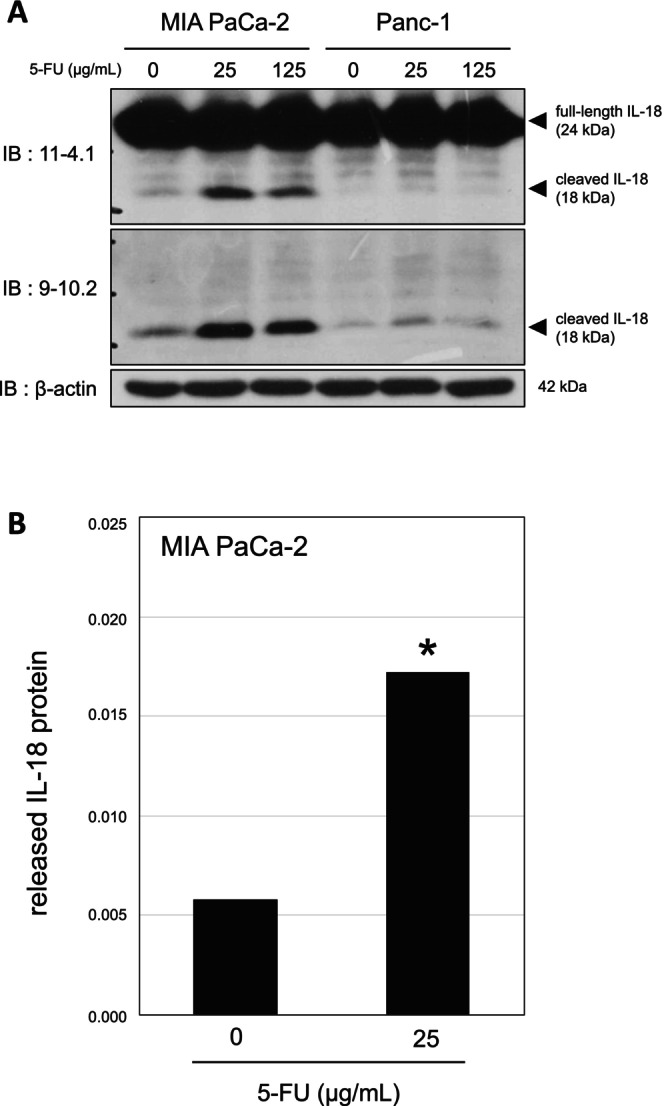
Induction of cleaved IL‐18 by 5‐FU treatment in human pancreatic cancer cell lines. (A) MIA PaCa‐2 and Panc‐1 cells were cultured in low‐nutrient culture medium and treated with 5‐FU at the indicated concentrations for 48 h. Whole‐cell lysates were analyzed by western blotting with anti‐IL‐18 mAbs. β‐Actin was used as a loading control. (B) Culture supernatants from MIA PaCa‐2 cells treated with 5‐FU for 48 h were analyzed by IL‐18 sandwich ELISA; absorbance (450–620 nm) is shown. *p < 0.05.

Capan‐2 cells were treated with 5‐FU in serum‐free RPMI 1640 culture medium for 48 h, after which whole‐cell lysates and culture supernatants were collected for western blotting and sandwich ELISA. Cleaved IL‐18 was detected using mAb 9‐10.2 (Figure S1A), and increased IL‐18 release into culture supernatants was observed after 5‐FU treatment (Figure S1B). We next examined additional pancreatic cancer cell lines. Cleaved IL‐18 was clearly induced in MIA PaCa‐2 cells and weakly induced in Panc‐1 cells (Figure 1A). Consistent with these results, IL‐18 release into the culture medium increased in 5‐FU–treated MIA PaCa‐2 cells (Figure 1B), indicating that this response is not restricted to Capan‐2 cells.

### 2.2 IL‐18 Activation Is Induced Independently of the NLRP3 Inflammasome

IL‐1β is another IL‐1 family cytokine that, like IL‐18, can be processed downstream of NLRP3 inflammasome activation (Man and Kanneganti 2015; Kelley et al. 2019). To assess whether IL‐1β was involved under our experimental conditions, we analyzed IL‐1β expression and processing in MIA PaCa‐2 and Panc‐1 cell lysates. Western blotting did not detect IL‐1β expression or cleavage in these lysates (Figure S2A). Unlike pro‐IL‐18, pro‐IL‐1β is not typically constitutively expressed and requires transcriptional induction by external signals (Zhu and Kanneganti 2017), suggesting that 5‐FU treatment under low‐nutrient conditions does not induce IL‐1β expression in these pancreatic cancer cells.

We next tested whether IL‐18 activation depended on the NLRP3 inflammasome. MIA PaCa‐2 cells were pretreated with the NLRP3 inhibitor MCC950 at various concentrations and then treated with 5‐FU in low‐nutrient culture medium for 48 h. MCC950 did not inhibit IL‐18 cleavage (Figure S2B). Together, these data suggest that IL‐18 cleavage in this system occurs independently of NLRP3 inflammasome activation.

### 2.3 Cleaved IL‐18 Is Enriched in Detached MIA PaCa‐2 Cells and Is Caspase‐1/4 Independent

**FIGURE 2:**
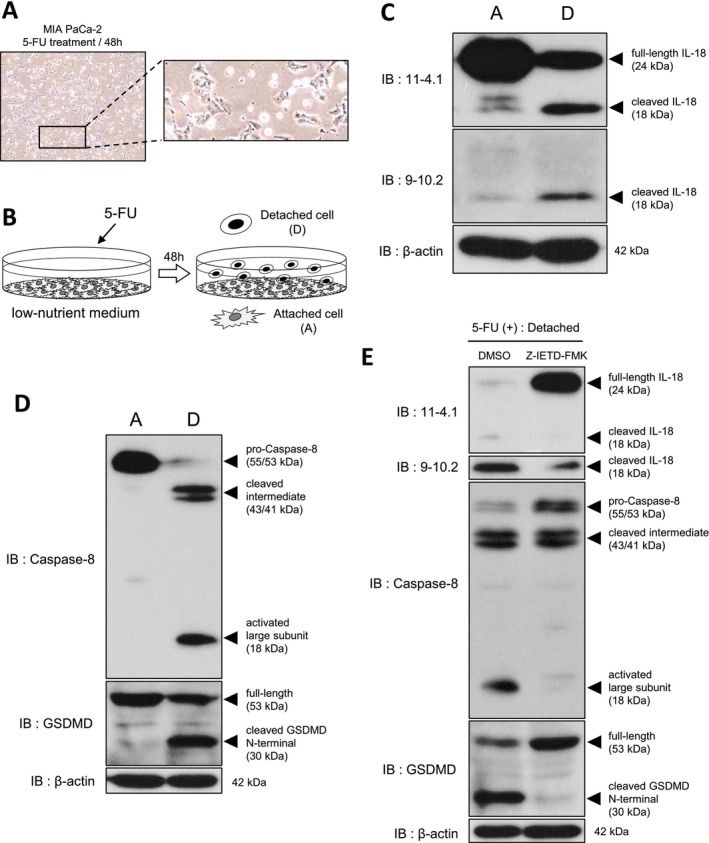
Cleavage of IL‐18 and GSDMD by caspase‐8 activation in detached MIA PaCa‐2 cells induced by nutrient starvation and 5‐FU treatment. (A) Representative image of MIA PaCa‐2 cells treated with 5‐FU (25 μg/mL) for 48 h in low‐nutrient medium (×10). An enlarged view of the indicated region is shown; both attached and detached cells are present. (B) Experimental overview. Attached and detached fractions are denoted [A] and [D], respectively. (C) Attached and detached cells after 5‐FU treatment (25 μg/mL, 48 h) were collected separately and analyzed by western blotting with anti‐IL‐18 mAbs. β‐Actin was used as a loading control. (D) Lysates from (C) were analyzed by western blotting with each anti‐Caspase‐8 and anti‐GSDMD antibody. β‐Actin was used as a loading control. (E) Cells were pretreated with DMSO or the caspase‐8 inhibitor Z‐IETD‐FMK (20 μM) and then treated with 5‐FU (100 μg/mL) in low‐nutrient medium. After 48 h, detached cells were collected and analyzed by western blotting with the indicated antibodies. β‐Actin was used as a loading control.

After 48 h of 5‐FU treatment in low‐nutrient culture medium, MIA PaCa‐2 cultures contained both attached cells and floating, detached cells (Figure 2A). To determine which fraction contributes most to IL‐18 activation, we collected attached and detached cells separately (Figure 2B) and performed western blotting. Full‐length IL‐18 decreased and cleaved IL‐18 increased markedly in detached cells compared with attached cells (Figure 2C), indicating that IL‐18 processing occurs predominantly in detached cells.

Because caspase‐1 (canonical) and caspase‐4 (non‐canonical) can mediate IL‐18 cleavage downstream of inflammasome signaling (Hayward et al. 2018), we examined caspase‐1 and caspase‐4 expression and activation in attached and detached cells. Western blotting showed no detectable differences in caspase‐1 or caspase‐4 expression or activation between the two fractions (Figure S3). Along with the lack of MCC950 sensitivity (Figure S2B), these results support a caspase‐1/4–independent mechanism of IL‐18 cleavage in detached MIA PaCa‐2 cells.

### 2.4 Caspase‐8 Mediates Pyroptosis Through Cleavage of GSDMD and IL‐18

Recent studies have shown that caspase‐8 can contribute to maturation of IL‐1β and IL‐18 (Bossaller et al. 2012) and can participate in pyroptosis (Gram et al. 2019). We therefore tested whether caspase‐8 is involved in IL‐18 cleavage in MIA PaCa‐2 cells. Pro‐caspase‐8 was readily detected in attached cells, whereas detached cells showed reduced pro‐caspase‐8 and increased cleaved intermediate and activated large subunit forms, consistent with caspase‐8 activation (Figure 2D).

We also assessed activation of GSDMD, a key executor of pyroptosis. The N‐terminal fragment of cleaved GSDMD forms membrane pores that facilitate cytokine release and drive pyroptotic cell death (Burdette et al. 2021). Full‐length GSDMD was present in both attached and detached cells, but the cleaved N‐terminal fragment was detected only in detached cells (Figure 2D), supporting the interpretation that detached cells are in pyroptosis.

To directly test the requirement for caspase‐8, we used the caspase‐8–specific inhibitor Z‐IETD‐FMK. MIA PaCa‐2 cells were pretreated with Z‐IETD‐FMK before 5‐FU treatment, and detached cells were collected for analysis. Z‐IETD‐FMK inhibited caspase‐8 processing (Figure 2E, middle) and markedly reduced cleavage of both IL‐18 and GSDMD (Figure 2E), indicating that caspase‐8 is a key mediator of IL‐18 and GSDMD cleavage in detached cells.

### 2.5 Activated Caspase‐8 Cleaves RIP1, Suggesting Suppression of Necroptosis

It was reported that caspase‐8 can inhibit necroptosis by cleaving RIP1, a central component of necroptotic signaling (Newton et al. 2019). Full‐length RIP1 was primarily detected in attached MIA PaCa‐2 cells, whereas detached cells showed reduced full‐length RIP1 and increased cleaved N‐terminal RIP1 fragment (Figure S4A). Moreover, Z‐IETD‐FMK inhibited RIP1 cleavage in detached cells (Figure S4B). These results suggest that caspase‐8 activation under nutrient starvation and 5‐FU treatment inhibits necroptosis and may promote pyroptosis.

### 2.6 Pyroptosis Is Induced by Nutrient Starvation Alone and Enhanced by 5‐FU Treatment

**FIGURE 3:**
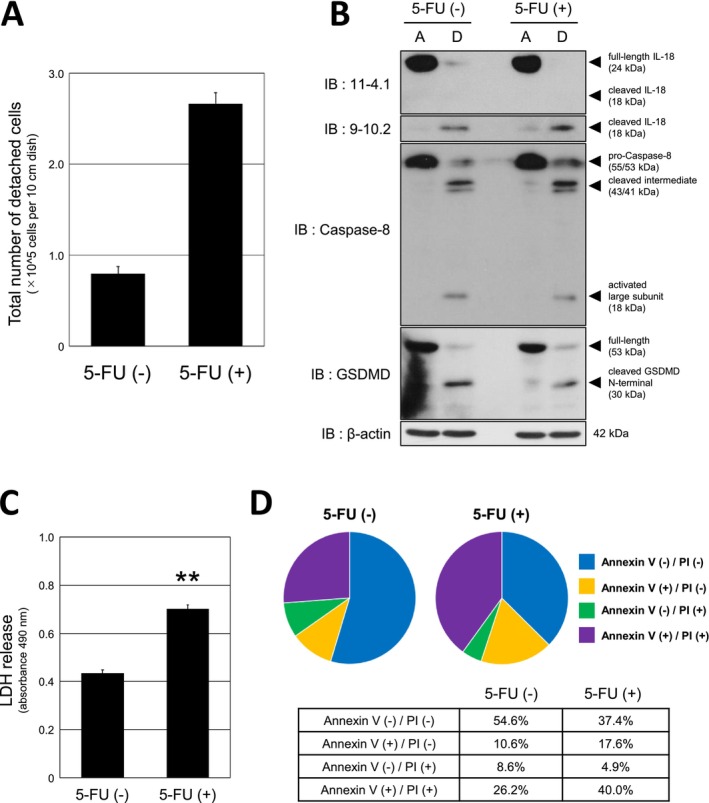
Analysis of detached cells emerged by low‐nutrient culture and influence of 5‐FU treatment. MIA PaCa‐2 cells were cultured in low‐nutrient medium with or without 5‐FU (25 μg/mL) for 48 h. (A) Detached cells from dish were collected and counted. Total number of detached cells present in 10 cm dish was shown as a bar graph. The experiment was performed twice. (B) Attached and detached cells were collected separately and analyzed by western blotting with the indicated antibodies. β‐Actin was used as a loading control. (C) Culture supernatants were analyzed by LDH release assay (*n* = 5). Absorbance was measured at 490 nm and summarized as a bar graph. **p < 0.01. (D) Detached cells were collected and stained with Annexin V‐FITC/propidium iodide (PI). Cells were analyzed using a flow cytometer and divided into four quadrants. The percentages for each subpopulation were shown in the pie chart and in table.

We noticed during experiments that a small fraction of MIA PaCa‐2 cells detached even in low‐nutrient culture medium without 5‐FU. The number of detached cells without 5‐FU was almost one‐third of cells with 5‐FU treatment (Figure 3A). We next compared IL‐18, caspase‐8, and GSDMD status in attached and detached fractions with and without 5‐FU. Notably, detached cells showed similar patterns of IL‐18 cleavage, caspase‐8 activation, and GSDMD cleavage regardless of 5‐FU treatment (Figure 3B). RIP1 cleavage was also detected in detached cells without 5‐FU (Figure S4C). These results suggested that pyroptosis of MIA PaCa‐2 cells was induced only by nutrient starvation.

Since the increase of detached cells was detected by 5‐FU treatment, we investigated the effects of 5‐FU on cells. LDH release was significantly higher with 5‐FU treatment (Figure 3C), supporting that 5‐FU affects cell damage. Furthermore, detached cells were examined by flow cytometric analysis about subpopulation status with the combination of Annexin V/Propidium Iodide (PI) staining. As shown in Figure 3D, a decrease of Annexin V/PI double negative cells and an increase of Annexin V/PI double positive cells were observed by 5‐FU treatment. Although Annexin V/PI double positive cells indicate any of late apoptotic/necroptotic/pyroptotic cells (Wang et al. 2021), these cells are not expected to be in necroptotic status due to RIP1 cleavage. Furthermore, the detection of cleavage of IL‐18 and GSDMD supports that detached cells were in pyroptosis. Together, these data indicate that nutrient starvation is sufficient to induce caspase‐8–associated pyroptotic signaling in detached MIA PaCa‐2 cells, while 5‐FU primarily increases the number of cells undergoing pyroptosis, leading to greater overall production of mature IL‐18.

### 2.7 Detached HCT116 Cells Exhibit IL‐18 and Caspase‐8 Activation Under Nutrient Starvation and 5‐FU Treatment

**FIGURE 4:**
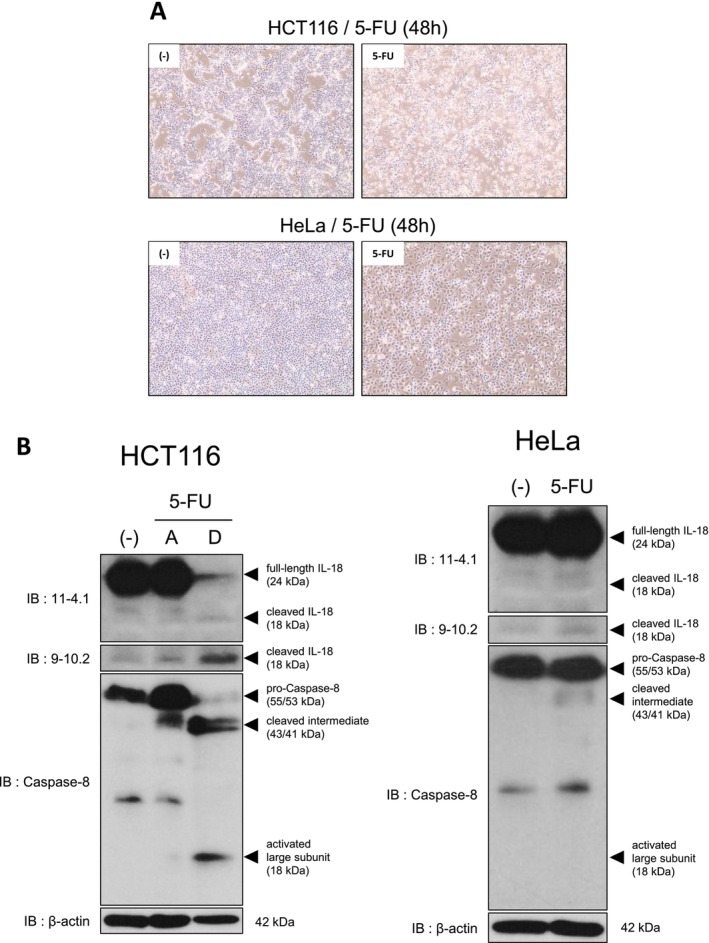
Cleaved IL‐18 induction by 5‐FU in cancer cells other than pancreatic cancer cells. (A) Representative images of HCT116 and HeLa cells treated with 5‐FU for 48 h in low‐nutrient culture medium (×10). 5‐FU was used at 10 μg/mL for HCT116 cells and 50 μg/mL for HeLa cells. Detached HCT116 cells were observed only after 5‐FU treatment. (B) HCT116 cells treated with 5‐FU were collected as attached or detached fractions; all other samples were collected as whole cells. Lysates were analyzed by western blotting with the indicated antibodies. β‐Actin was used as a loading control.

To examine whether IL‐18 activation under starvation and 5‐FU treatment occurs in cancer cell types beyond pancreatic cancer, we tested human colon cancer HCT116 cells and human cervical cancer HeLa cells. In low‐nutrient culture medium, HCT116 cells detached after 5‐FU treatment (Figure 4A upper right), whereas little to no detachment was observed without 5‐FU (Figure 4A, upper left). Accordingly, for HCT116 cells we collected attached and detached fractions after 5‐FU treatment, whereas without 5‐FU we prepared whole‐cell lysates. In contrast, HeLa cells showed growth arrest with cell enlargement after 5‐FU treatment but exhibited little to no detachment under either condition (Figure 4A, lower), and therefore whole‐cell lysates were analyzed.

Western blotting revealed IL‐18 cleavage and caspase‐8 activation only in detached HCT116 cells after 5‐FU treatment (Figure 4B). These findings suggest that caspase‐8–associated IL‐18 activation in detached cells under nutrient starvation and 5‐FU treatment is not limited to pancreatic cancer cells.

### 2.8 Other Pancreatic Cancer Therapeutics Do Not Enhance IL‐18 Activation

**FIGURE 5:**
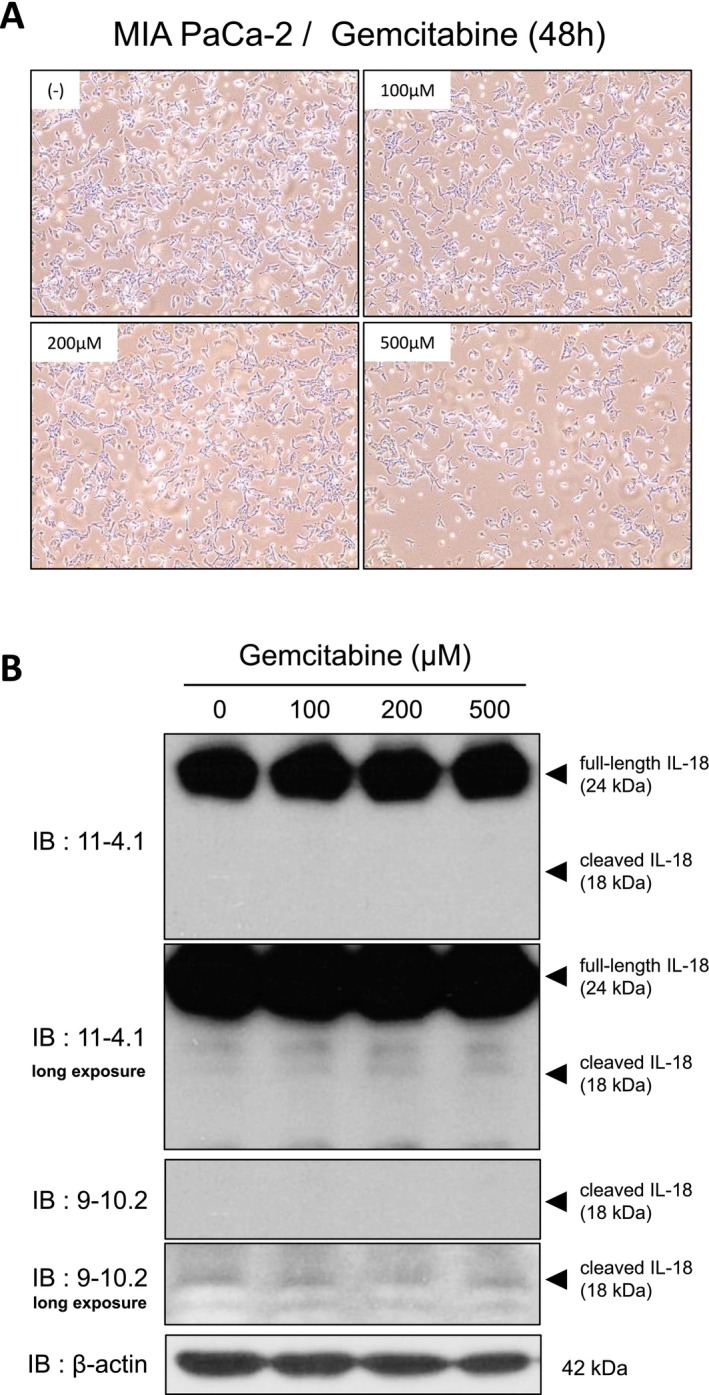
Gemcitabine does not induce cleaved IL‐18 in MIA PaCa‐2 cells under low‐nutrient conditions. (A) Representative images of MIA PaCa‐2 cells treated with gemcitabine for 48 h in low‐nutrient culture medium (×10). Although dose‐dependent growth arrest was observed, little to no detachment occurred. (B) Whole‐cell lysates were analyzed by western blotting with anti‐IL‐18 mAbs. β‐Actin was used as a loading control.

We next tested whether standard pancreatic cancer therapeutics besides 5‐FU promote IL‐18 maturation. MIA PaCa‐2 cells were treated with gemcitabine (up to 500 μM) in low‐nutrient culture medium. Although gemcitabine induced dose‐dependent growth arrest, it caused little to no cell detachment (Figure 5A). Western blotting did not detect cleaved IL‐18 in gemcitabine‐treated cells (Figure 5B), indicating that gemcitabine does not induce IL‐18 activation under these conditions.

We also examined components of the FOLFIRINOX regimen (5‐FU, levofolinate, irinotecan, and oxaliplatin) (Ettrich and Seufferlein 2021). Levofolinate alone did not induce IL‐18 cleavage, and co‐treatment with 5‐FU did not enhance IL‐18 processing (Figure S5A). Irinotecan or oxaliplatin alone induced IL‐18 cleavage compared with untreated controls, but less strongly than 5‐FU (Figure S5B). Co‐treatment of 5‐FU with oxaliplatin yielded cleaved IL‐18 levels similar to 5‐FU alone, suggesting that oxaliplatin does not augment IL‐18 activation. In contrast, co‐treatment of 5‐FU with irinotecan appeared to reduce cleaved IL‐18 compared with 5‐FU alone (Figure S5B), although the mechanism remains unclear. Treatment with all four drugs in combination did not enhance cleaved IL‐18 and instead reduced it in a dose‐dependent manner (Figure S5C). We speculate that higher‐dose combinations may induce rapid cell death distinct from pyroptosis, but further investigation is needed. Overall, IL‐18 activation induced by 5‐FU was not enhanced by additional pancreatic cancer drugs, supporting a key role for 5‐FU in promoting pyroptotic cell death with IL‐18 cleavage.

## 3 Discussion

**FIGURE 6:**
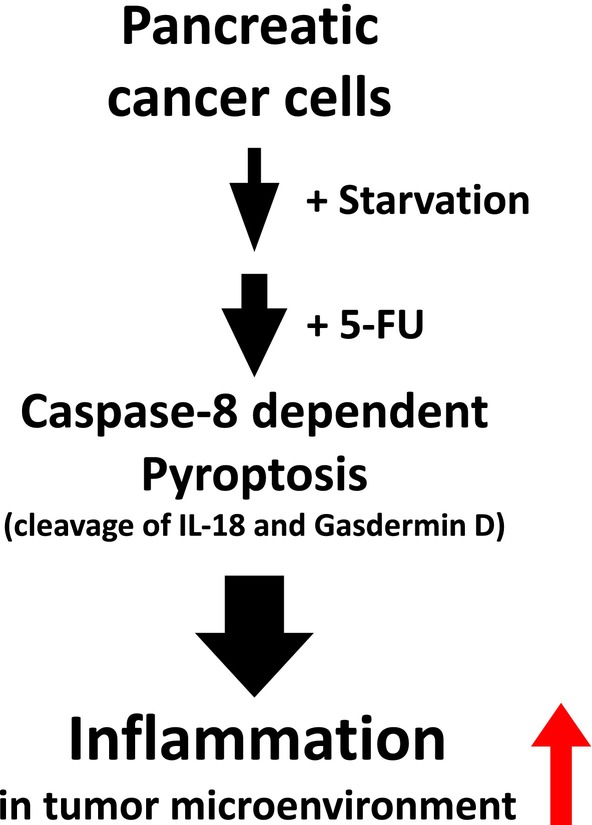
Proposed model of pyroptosis induction in pancreatic cancer cells. Nutrient starvation induces caspase‐8–dependent cell pyroptosis accompanied by IL‐18 and GSDMD cleavage. 5‐FU increases the number of cells undergoing pyroptosis. Increased release of active IL‐18 can ultimately lead to chronic inflammation in the tumor environment.

In this study, we investigated the mechanism by which pancreatic cancer cells generate mature, active IL‐18 under low‐nutrient conditions and in response to 5‐FU treatment (Figure 6). Carbone and colleagues first reported that pancreatic cancer Capan‐2 cells produce bioactive IL‐18 after 5‐FU treatment (Carbone et al. 2005). Using IL‐18 monoclonal antibodies that we developed (Nariai et al. 2019), we confirmed that cleaved IL‐18 is induced in Capan‐2 cells and also in MIA PaCa‐2 cells. Because pancreatic tumor interiors are characterized by hypoxia and nutrient limitation (Kamphorst et al. 2015), a similar phenomenon may occur in vivo during 5‐FU‐based therapy.

What might be the impact of therapy‐induced IL‐18 release on the tumor microenvironment? IL‐18 can enhance anti‐tumor immunity by promoting interferon‐γ production from immune cells, including T cells and natural killer cells (Okamura et al. 1995; Chaix et al. 2008). Conversely, IL‐18 has also been reported to support tumor progression by upregulating angiogenesis (e.g., via VEGF induction), enhancing metastasis (e.g., via induction of adhesion molecules and matrix metalloproteases), and promoting immunosuppressive myeloid‐derived suppressor cells (MDSCs) (Palma et al. 2013; Lim et al. 2014). In pancreatic cancer, higher IL‐18 expression in tumor tissues correlates with increased metastasis and shorter survival (Guo et al. 2016). Thus, while 5‐FU has anti‐tumor effects, it may also influence tumor progression through induction of mature IL‐18 production. Combined strategies incorporating 5‐FU and IL‐18 inhibition at tumor sites may improve therapeutic outcomes, although this hypothesis will require further validation.

By separating attached and detached cell fractions, we found that IL‐18 cleavage occurs predominantly in detached cells after 5‐FU treatment under low‐nutrient culture conditions (Figure 2). These detached cells also showed GSDMD cleavage, supporting that cells are undergoing pyroptosis. Canonical pyroptosis is driven by inflammasomes such as NLRP3, which recruit ASC and pro‐caspase‐1 and lead to caspase‐1 activation, cleavage of IL‐1β/IL‐18, GSDMD processing, and membrane pore formation (Man and Kanneganti 2015; Hayward et al. 2018; Kelley et al. 2019). In non‐canonical pyroptosis, caspase‐4/5 (humans) or caspase‐11 (mice) is activated by cytosolic LPS and directly cleaves GSDMD; this pathway can also promote NLRP3 activation and caspase‐1–dependent cytokine maturation through incompletely defined mechanisms (Downs et al. 2020; Matikainen et al. 2020). In our experiments, however, IL‐18 cleavage was not inhibited by an NLRP3 inhibitor (Figure S2B), IL‐1β was not induced (Figure S2A), and caspase‐1/4 activation did not differ between attached and detached cells (Figure S3). Instead, we observed caspase‐8–dependent cleavage of IL‐18 and GSDMD (Figure 2E).

Caspase‐8 is classically known as an initiator of apoptosis, but accumulating evidence supports broader roles for caspase‐8 in inflammatory programmed cell death pathways, including pyroptosis and necroptosis, as part of “PANoptosis” (Malireddi et al. 2019; Chowdhury and Hasan 2026). Several studies have shown that caspase‐8 can cleave IL‐18 and GSDMD directly (Bossaller et al. 2012; Sarhan et al. 2018), consistent with our observations. The upstream signals that activate caspase‐8 under nutrient starvation and 5‐FU treatment remain unclear and warrant further investigation. Carbone et al. reported that a caspase‐1 inhibitor reduced IL‐18 maturation and secretion in Capan‐2 cells after 5‐FU treatment (Carbone et al. 2005), but caspase‐8 activation was detected in detached Capan‐2 cells in our experiments (Data not shown). Differences in experimental conditions and analysis strategy—particularly our focus on detached cells—may contribute to these divergent findings.

Importantly, we found that nutrient starvation alone is sufficient to induce pyroptosis in MIA PaCa‐2 cells, and 5‐FU treatment contributed to an increased number of detached cells undergoing pyroptosis (Figure 3A). The relationship between starvation and pyroptosis in cancer cells remains poorly defined. Starvation has been reported to promote pyroptosis in myocardial tissues and cells (Wang et al. 2018), but mechanisms in tumor cells require further clarification. On the other hand, almost half of the detached cells in low‐nutrient culture and one‐third of the detached cells in low‐nutrient culture with 5‐FU treatment showed double negative staining of Annexin V/PI (Figure 3D). We currently do not have an answer regarding the state of these subpopulations, but based on the results of the immunoblot analysis, it is suspected that they are in a state of progressing pyroptosis, and further analysis is needed.

We also observed a marked reduction of pro‐IL‐18 in detached cells under starvation with or without 5‐FU (Figure 3B). This reduction was canceled by caspase‐8–specific inhibitor Z‐IETD‐FMK treatment (Figure 2E), suggesting that caspase‐8 is deeply involved in this phenomenon at least under 5‐FU treatment. There is a possibility that caspase‐8 inactivation inhibits both cleavage of IL‐18 and formation of GSDMD pores, resulting in a large amount of pro‐IL‐18 being present within MIA PaCa‐2 cells. Meanwhile, cell detachment also occurred under caspase‐8 inhibited conditions (Figure 2E). This fact suggests that these cells may have detached for reasons other than pyroptosis, requiring further investigation. On the other hand, it was reported recently that 5‐FU induces apoptosis in hepatocellular carcinoma under nutritional deprived condition (Dutta et al. 2024). The effect of 5‐FU in low‐nutrient condition may differ between pancreatic and liver cancer.

Finally, we examined whether this mechanism extends beyond pancreatic cancer. Detached HCT116 colorectal cancer cells exhibited IL‐18 cleavage and caspase‐8 activation under low‐nutrient culture conditions and 5‐FU treatment (Figure 4B), suggesting that this response can occur in other cancer types. Notably, in colorectal tumors, higher IL‐18 expression has been reported to correlate positively with overall survival (Feng et al. 2020), raising the possibility that the consequences of IL‐18 induction may differ by tumor context. HeLa cells did not detach under our conditions, although whole‐cell lysates showed slight induction of cleaved IL‐18 and cleaved intermediate caspase‐8 (Figure 4B), suggesting that adjustments to experimental conditions might reveal pyroptotic features. We also did not observe induction of pyroptosis in normal human peripheral blood lymphocytes under these conditions (Data not shown). Further work is needed to determine which normal cell types, if any, undergo similar responses.

In conclusion, we elucidated a mechanism of caspase‐8–dependent pyroptotic cell death with cleavage of IL‐18 and GSDMD induced by nutrient starvation in pancreatic cancer cells. We further found that 5‐FU treatment enhances cell detachment and the extent of cell pyroptosis. These findings raise the possibility that, while 5‐FU exerts anticancer effects, it may also promote inflammatory signaling through IL‐18 activation in nutrient‐restricted tumor microenvironments. In pancreatic cancer, where higher IL‐18 expression in tumor tissue is associated with poor prognosis, strategies that combine 5‐FU therapy with local IL‐18 inhibition may warrant further exploration.

## 4 Experimental Procedures

### 4.1 Cell Lines and Reagents

Human pancreatic cancer cell lines (MIA PaCa‐2 and Panc‐1), human colorectal cancer cell line (HCT116), and human cervical cancer cell line (HeLa) were purchased from the American Type Culture Collection. Human pancreatic cancer cell line Capan‐2 was kindly provided by Dr. Y. Honma (Shimane University). MIA PaCa‐2, HCT116, and HeLa cells were maintained in DMEM (Nissui, Japan), and Capan‐2 cells were maintained in RPMI 1640 (Thermo Fisher Scientific, Waltham, MA, USA). Both media were supplemented with 10% fetal bovine serum (Sigma‐Aldrich, St. Louis, MO, USA). Cells were cultured at 37°C in a humidified atmosphere containing 5% CO_2_. All cell lines were confirmed that mycoplasma were free throughout the study period.

5‐Fluorouracil (5‐FU) and irinotecan were obtained from Towa Yakuhin (Japan). Oxaliplatin and calcium levofolinate hydrate were obtained from Nihon Kayaku (Japan). Gemcitabine was obtained from Pfizer (Japan). The caspase‐8 inhibitor Z‐IETD‐FMK was obtained from R&D Systems (USA). The NLRP3 inflammasome inhibitor MCC950 was obtained from AdipoGen (Switzerland).

### 4.2 Antibodies

Anti‐human IL‐18 monoclonal antibodies (mAbs) 11‐4.1 and 9‐10.2 were generated in our laboratory (Nariai et al. 2019). Anti‐human IL‐1β mAb 7‐6.1 and anti‐human caspase‐1 mAb 12‐6.2 were also generated in our laboratory. Anti‐caspase‐4 rabbit polyclonal antibody (11856‐1‐AP) was purchased from Proteintech (USA). Anti‐caspase‐8 antibody (M032‐3) was purchased from MBL (Japan). Anti‐gasdermin D antibody (NBP2‐33422) was purchased from Novus Biologicals (USA). Anti‐β‐actin antibody (AC‐15) was purchased from Sigma‐Aldrich (USA). A necroptosis antibody sampler kit (#98110), including anti‐RIP1 antibody, was purchased from Cell Signaling Technology (USA).

### 4.3 Preparation of Cell Lysates and Western Blotting

Cancer cells were seeded in 10 cm dishes. The following day, culture medium was replaced with Opti‐MEM (as a replacement for DMEM) or serum‐free RPMI 1640, and anticancer drugs were added for 48 h at the indicated concentrations (5‐FU: up to ~1000 μg/mL; gemcitabine: up to ~500 μM; levofolinate: up to ~25 μg/mL; irinotecan: up to ~100 μg/mL; oxaliplatin: up to ~25 μg/mL). For experiments using Z‐IETD‐FMK (20 μM) or MCC950 (up to ~10 μM), inhibitors were added 1 h before anticancer drug treatment. After treatment, cell lysates were prepared from (i) mixed attached and detached cells (whole cells), (ii) detached cells only, or (iii) attached cells only. Cells were lysed in SDS sample buffer (125 mM Tris‐HCl, pH 6.8, 5% glycerol, 4% SDS, 0.005% bromophenol blue, and 10% 2‐mercaptoethanol). Lysate protein concentrations were determined using XL‐Bradford reagent (KY‐1030; Aproscience, Japan), and equal volumes of each lysate were subjected to SDS–polyacrylamide gel electrophoresis (PAGE). Western blotting was performed as described previously (Guo et al. 2016). After blocking, membranes were incubated with the indicated primary antibodies (1:1000) for 1.5 h at room temperature and then incubated with HRP‐conjugated secondary antibodies (anti‐mouse or anti‐rabbit IgG; 1:2000) for 20 min. Antibody binding was detected using Western Lightning Plus‐ECL reagents (PerkinElmer, USA).

### 4.4 Enzyme‐Linked Immunosorbent Assay (ELISA)

Released IL‐18 in culture supernatants after 5‐FU treatment was measured using a human IL‐18 ELISA kit (7620; MBL, Nagoya, Japan) according to the manufacturer's instructions. Briefly, MIA PaCa‐2 or Capan‐2 cells were cultured with or without 5‐FU in Opti‐MEM or serum‐free RPMI 1640 for 48 h, and collected supernatants were used for ELISA. Absorbance was measured at 450 nm with a reference wavelength of 620 nm.

### 4.5 LDH Release Assay

Lactate dehydrogenase (LDH) release was assessed using the Cytotoxicity LDH Assay Kit‐WST (Dojindo, Japan) to evaluate cell death, including pyroptosis‐associated membrane damage. Cells were cultured in flat‐bottom 96‐well plates with or without 5‐FU for 48 h, and the assay was performed according to the manufacturer's instructions. Absorbance was measured at 490 nm.

### 4.6 Annexin V‐FITC/PI‐Staining Assay

Detached MIA PaCa‐2 cells were collected and counted, then 2 × 10^5^ cells were suspended in 1 × Biniding buffer. After centrifugation step, cells were suspended in small volume of 1 × Biniding buffer and stained with 5 μL of Annexin V‐FITC (BioLegend, USA) and 2 μL of 1 mg/mL PI stock solution (Sigma‐Aldrich, USA). After 15 min incubation on ice, cells were filtrated with cell strainer and analyzed using CytoFLEX S (Beckman, USA). In total 10,000 or 30,000 cells were analyzed per measurement, and obtained data was analyzed using CytExpert 2.4 software (Beckman, USA).

### 4.7 Statistical Analysis

Student's *t*‐test (two‐group comparisons) was used for IL‐18 ELISA, quantification of detached‐cell lysate protein amounts, and LDH release assays. A *p* value < 0.05 was considered statistically significant.

## Author Contributions

**Hiroki Kamino:** conceptualization, investigation, data curation, formal analysis, writing – original draft, writing – review and editing, funding acquisition. **Yuko Nariai:** investigation, data curation, formal analysis, writing – review and editing, funding acquisition. **Takeshi Urano:** conceptualization, investigation, formal analysis, data curation, writing – review and editing, funding acquisition.

## Funding

This work was supported by JSPS KAKENHI (20K07635 to H.K.), by AMED (B8F2025A01 to Y.N.), and by the Shimane University “SUIGAN Project” (to T.U.).

## Ethics Statement

The authors have nothing to report.

## Conflicts of Interest

T.U. is employed by Shimane University and is currently co‐founder and Chief Medical & Scientific Officer of mAbProtein, a biotech company focusing on the development and commercial utilization of mAbs for inflammation research, diagnosis, and treatment. Y.N. is also employed by Shimane University and, through the cross‐appointment system, receives a salary from mAbProtein Co. Ltd. H.K. declare no conflicts of interest.

## Supporting information


**Figure S1:** Induction of cleaved IL‐18 by 5‐FU treatment in Capan‐2 cells. (A) Capan‐2 cells were cultured in serum‐free RPMI 1640 and treated with 5‐FU at the indicated concentrations for 48 h. Whole‐cell lysates were analyzed by western blotting. β‐actin was used as a loading control. (B) Culture supernatants were analyzed by IL‐18 sandwich ELISA; absorbance (450–620 nm) is shown as a bar graph. **p < 0.01.

**Figure S2:** Evidence that IL‐18 is cleaved in an NLRP3 inflammasome‐independent manner. (A) Lysates from MIA PaCa‐2 and Panc‐1 cells used in Figure 1A were analyzed by western blotting with the in‐house anti‐IL‐1β mAb 7‐6.1. Recombinant cleaved IL‐1β was used as a positive control. (B) MIA PaCa‐2 cells were pretreated with the NLRP3 inhibitor MCC950 at the indicated concentrations and then treated with 5‐FU (25 μg/mL) in low‐nutrient medium for 48 h. Whole‐cell lysates were analyzed by western blotting with anti‐IL‐18 mAbs. β‐actin was used as a loading control.

**Figure S3:** Evidence that IL‐18 cleavage occurs independently of caspase‐1/4. Attached and detached MIA PaCa‐2 cell lysates used in Figure 2C,D were analyzed by western blotting with anti‐caspase‐1 and anti‐caspase‐4 antibodies. Attached and detached fractions are denoted [A] and [D], respectively. Estimated molecular weights of pro‐caspase‐1 and pro‐caspase‐4 are 45.2 and 43.3 kDa, respectively. β‐actin was used as a loading control (same loading control as in Figure 2D).

**Figure S4:** Cleavage of RIP1 in MIA PaCa‐2 cells treated with or without 5‐FU under low‐nutrient culture condition. (A) Lysates from Figure 2D were analyzed by western blotting with anti‐RIP1 antibody. β‐actin was used as a loading control (same loading control as in Figure 2D). (B) Lysates from Figure 2E were analyzed by western blotting with anti‐RIP1 antibody. β‐actin was used as a loading control (same loading control as in Figure 2E). (C) Lysates from Figure 3B were analyzed by western blotting with anti‐RIP1 antibody. Attached and detached fractions are denoted [A] and [D], respectively. β‐actin was used as a loading control (same loading control as in Figure 3B).

**Figure S5:** Limited influence of FOLFIRINOX components on IL‐18 induction in MIA PaCa‐2 cells. (A) MIA PaCa‐2 cells were treated with 5‐FU and/or levofolinate at the indicated concentrations for 48 h in low‐nutrient culture medium. Whole‐cell lysates were analyzed by western blotting with anti‐IL‐18 mAbs. β‐actin was used as a loading control. (B) MIA PaCa‐2 cells were treated with irinotecan (20 μg/mL) or oxaliplatin (2.5 μg/mL) alone or in combination with 5‐FU (25 μg/mL) for 48 h in low‐nutrient culture medium. Whole‐cell lysates were analyzed by western blotting with anti‐IL‐18 mAbs. β‐actin was used as a loading control. (C) MIA PaCa‐2 cells were treated with the four‐drug combination at the indicated concentrations for 48 h in low‐nutrient culture medium. Whole‐cell lysates were analyzed by western blotting with anti‐IL‐18 mAbs. β‐actin was used as a loading control.

## Data Availability

The data that support the findings of this study are available from the corresponding author upon reasonable request.
