# Supplementary material for: Molecular Mechanism of Caspase‐8–Dependent Interleukin‐18 Activation in Pancreatic Cancer Cells Induced by 5‐Fluorouracil and Nutrient Starvation
Source: Genes Cells. 2026 Apr 6;31(3):e70111. doi: 10.1111/gtc.70111 (PMC13051528; doi:10.1111/gtc.70111)
Supplement: Supplementary file 1 — Figure S1: Induction of cleaved IL‐18 by 5‐FU treatment in Capan‐2 cells. (A) Capan‐2 cells were cultured in serum‐free RPMI 1640 and treated with 5‐FU at the indicated concentrations for 48 h. Whole‐cell lysates were analyzed by western blotting. β‐actin was used as a loading control. (B) Culture supernatants were analyzed by IL‐18 sandwich ELISA; absorbance (450–620 nm) is shown as a bar graph. **p < 0.01. [file GTC-31-0-s002.pptx]

## Slide 1
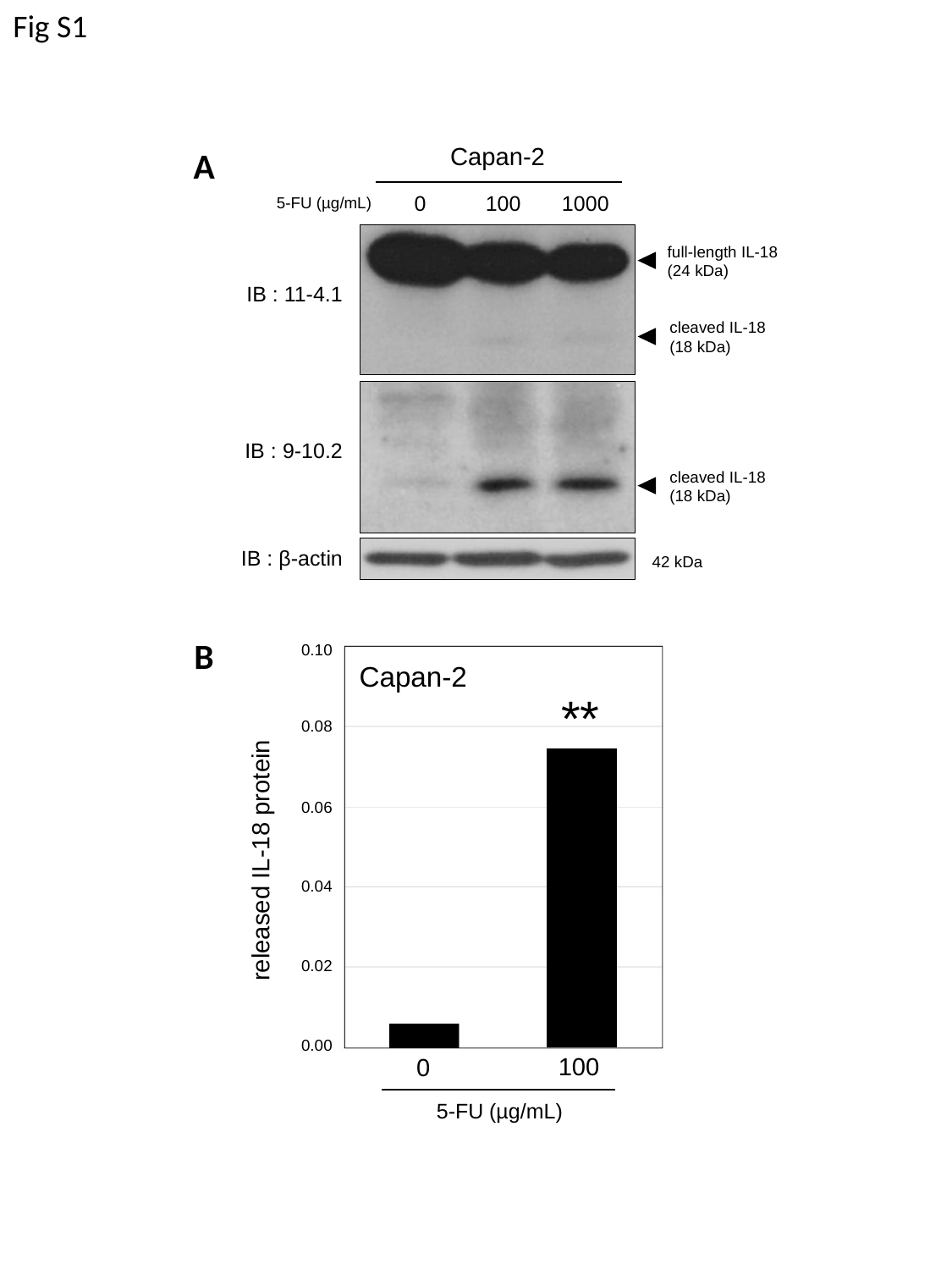

Fig S1
Capan-2
A
0
100
1000
5-FU (µg/mL)
full-length IL-18
(24 kDa)
IB : 11-4.1
cleaved IL-18
(18 kDa)
IB : 9-10.2
cleaved IL-18
(18 kDa)
IB : β-actin
42 kDa
B
0.10
Capan-2
**
0.08
0.06
released IL-18 protein
0.04
0.02
0.00
100
0
5-FU (µg/mL)
