# Supplementary material for: Molecular Mechanism of Caspase‐8–Dependent Interleukin‐18 Activation in Pancreatic Cancer Cells Induced by 5‐Fluorouracil and Nutrient Starvation
Source: Genes Cells. 2026 Apr 6;31(3):e70111. doi: 10.1111/gtc.70111 (PMC13051528; doi:10.1111/gtc.70111)
Supplement: Supplementary file 2 — Figure S2: Evidence that IL‐18 is cleaved in an NLRP3 inflammasome‐independent manner. (A) Lysates from MIA PaCa‐2 and Panc‐1 cells used in Figure 1A were analyzed by western blotting with the in‐house anti‐IL‐1β mAb 7‐6.1. Recombinant cleaved IL‐1β was used as a positive control. (B) MIA PaCa‐2 cells were pretreated with the NLRP3 inhibitor MCC950 at the indicated concentrations and then treated with 5‐FU (25 μg/mL) in low‐nutrient medium for 48 h. Whole‐cell lysates were analyzed by western blotting with anti‐IL‐18 mAbs. β‐actin was used as a loading control. [file GTC-31-0-s004.pptx]

## Slide 1
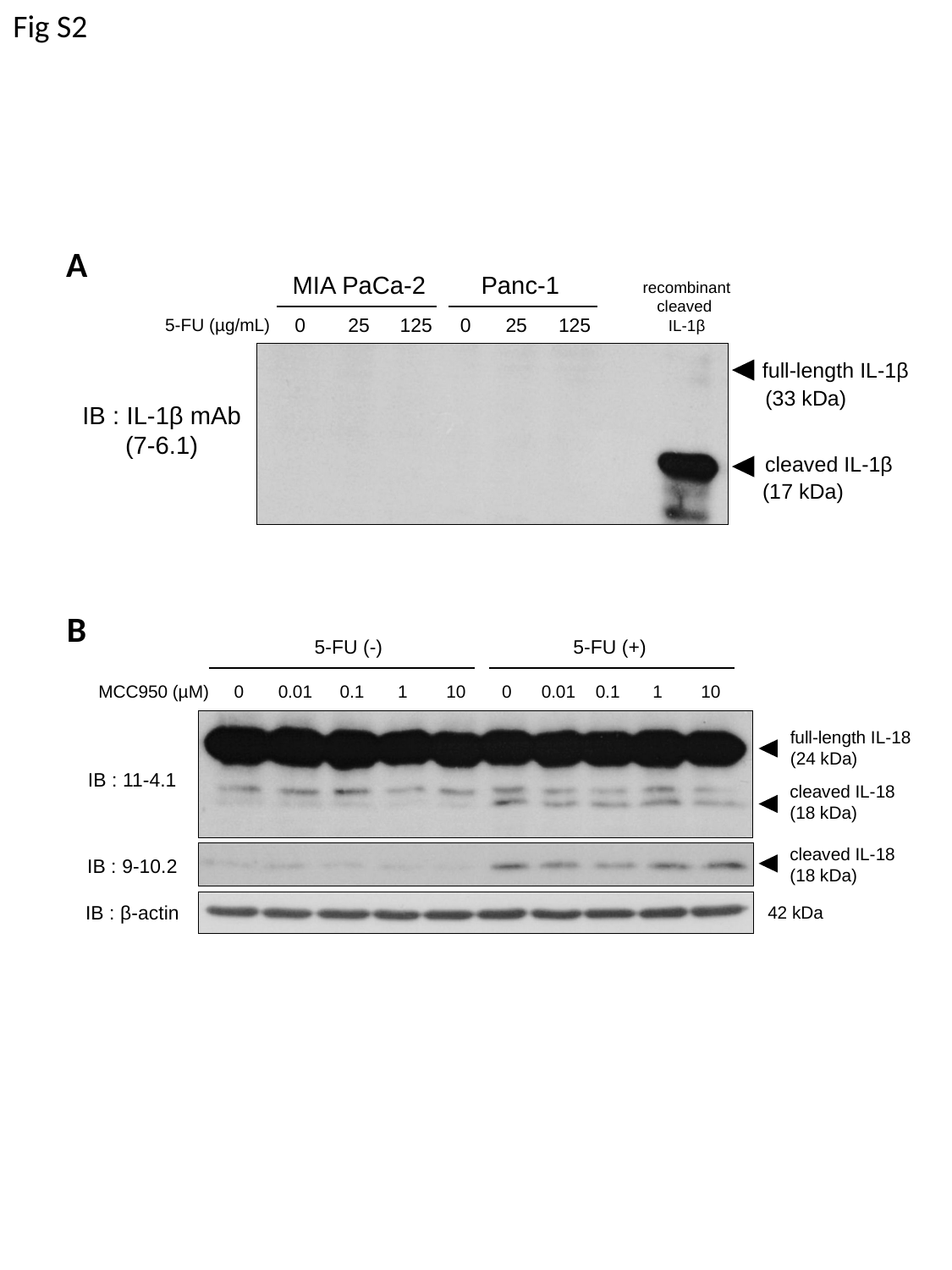

Fig S2
A
MIA PaCa-2
Panc-1
recombinant
cleaved
IL-1β
25
125
125
0
0
25
5-FU (µg/mL)
full-length IL-1β
(33 kDa)
IB : IL-1β mAb
(7-6.1)
cleaved IL-1β
(17 kDa)
B
5-FU (-)
5-FU (+)
MCC950 (µM)
0
0.01
0.1
1
10
0
0.01
0.1
1
10
full-length IL-18
(24 kDa)
IB : 11-4.1
cleaved IL-18
(18 kDa)
cleaved IL-18
(18 kDa)
IB : 9-10.2
IB : β-actin
42 kDa
