# Supplementary figures and images for: Molecular Mechanism of Caspase‐8–Dependent Interleukin‐18 Activation in Pancreatic Cancer Cells Induced by 5‐Fluorouracil and Nutrient Starvation
Source: Genes Cells. 2026 Apr 6;31(3):e70111. doi: 10.1111/gtc.70111 (PMC13051528; doi:10.1111/gtc.70111)

## Slide 1
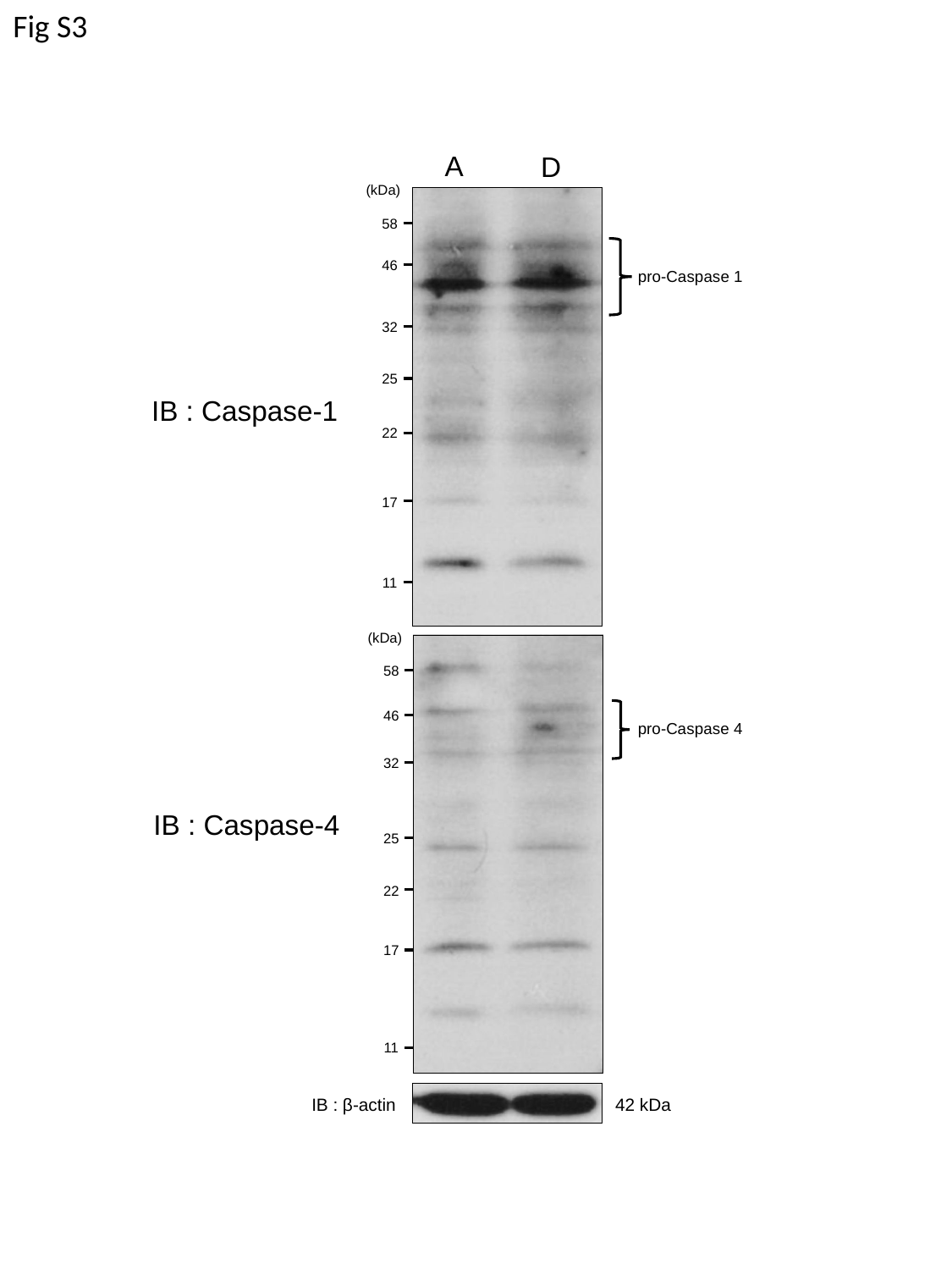

Fig S3
A
D
(kDa)
58
46
pro-Caspase 1
32
25
IB : Caspase-1
22
17
11
(kDa)
58
46
pro-Caspase 4
32
IB : Caspase-4
25
22
17
11
IB : β-actin
42 kDa

Supplement: Supplementary file 3 — Figure S3: Evidence that IL‐18 cleavage occurs independently of caspase‐1/4. Attached and detached MIA PaCa‐2 cell lysates used in Figure 2C,D were analyzed by western blotting with anti‐caspase‐1 and anti‐caspase‐4 antibodies. Attached and detached fractions are denoted [A] and [D], respectively. Estimated molecular weights of pro‐caspase‐1 and pro‐caspase‐4 are 45.2 and 43.3 kDa, respectively. β‐actin was used as a loading control (same loading control as in Figure 2D). [file GTC-31-0-s003.pptx]
