# Supplementary material for: Molecular Mechanism of Caspase‐8–Dependent Interleukin‐18 Activation in Pancreatic Cancer Cells Induced by 5‐Fluorouracil and Nutrient Starvation
Source: Genes Cells. 2026 Apr 6;31(3):e70111. doi: 10.1111/gtc.70111 (PMC13051528; doi:10.1111/gtc.70111)
Supplement: Supplementary file 4 — Figure S4: Cleavage of RIP1 in MIA PaCa‐2 cells treated with or without 5‐FU under low‐nutrient culture condition. (A) Lysates from Figure 2D were analyzed by western blotting with anti‐RIP1 antibody. β‐actin was used as a loading control (same loading control as in Figure 2D). (B) Lysates from Figure 2E were analyzed by western blotting with anti‐RIP1 antibody. β‐actin was used as a loading control (same loading control as in Figure 2E). (C) Lysates from Figure 3B were analyzed by western blotting with anti‐RIP1 antibody. Attached and detached fractions are denoted [A] and [D], respectively. β‐actin was used as a loading control (same loading control as in Figure 3B). [file GTC-31-0-s005.pptx]

## Slide 1
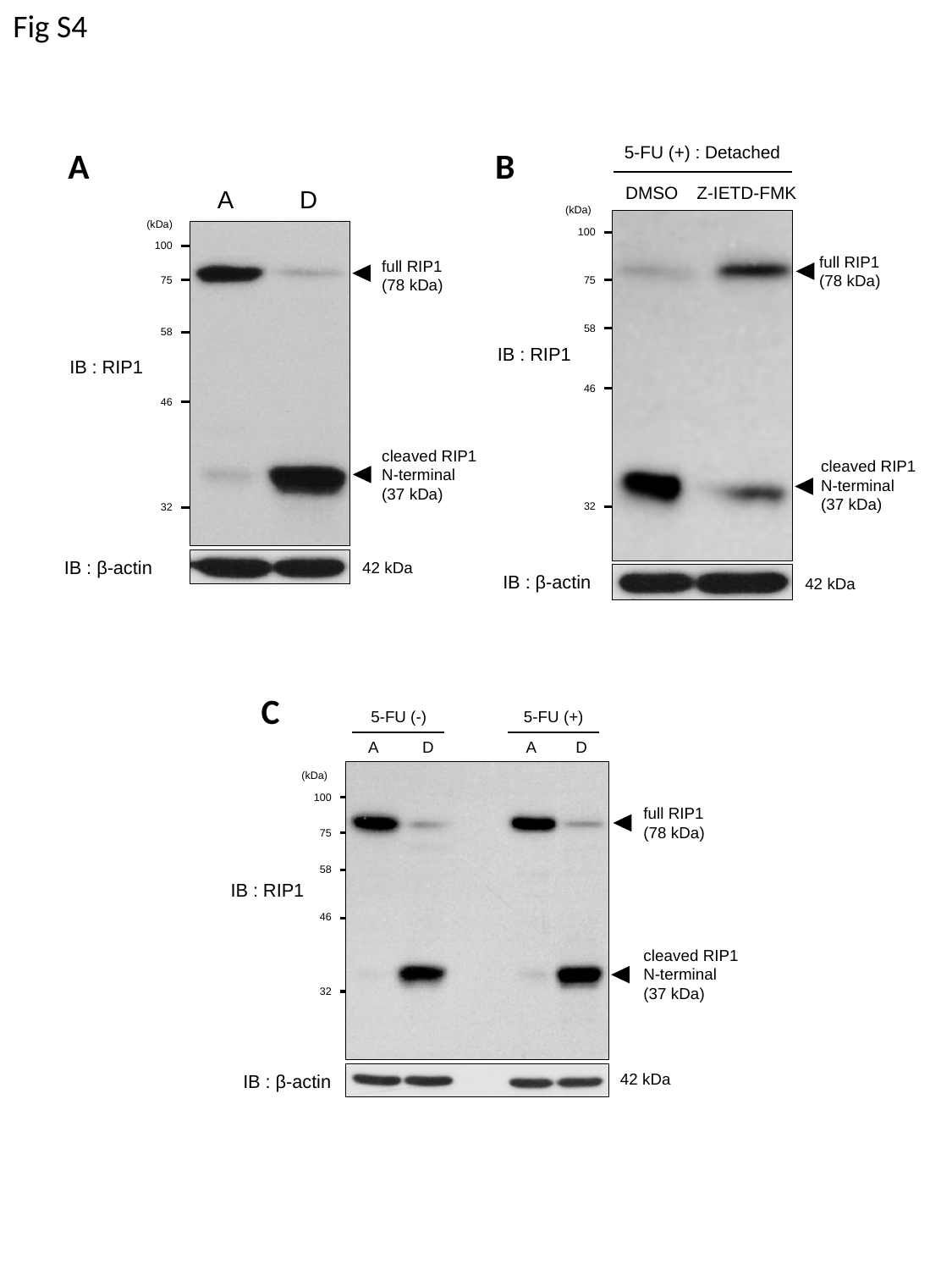

Fig S4
5-FU (+) : Detached
A
B
DMSO
Z-IETD-FMK
D
A
(kDa)
(kDa)
100
100
full RIP1
(78 kDa)
full RIP1
(78 kDa)
75
75
58
58
IB : RIP1
IB : RIP1
46
46
cleaved RIP1
N-terminal
(37 kDa)
cleaved RIP1
N-terminal
(37 kDa)
32
32
IB : β-actin
42 kDa
IB : β-actin
42 kDa
C
5-FU (-)
5-FU (+)
A
D
A
D
(kDa)
100
full RIP1
(78 kDa)
75
58
IB : RIP1
46
cleaved RIP1
N-terminal
(37 kDa)
32
42 kDa
IB : β-actin
