# Supplementary material for: Molecular Mechanism of Caspase‐8–Dependent Interleukin‐18 Activation in Pancreatic Cancer Cells Induced by 5‐Fluorouracil and Nutrient Starvation
Source: Genes Cells. 2026 Apr 6;31(3):e70111. doi: 10.1111/gtc.70111 (PMC13051528; doi:10.1111/gtc.70111)
Supplement: Supplementary file 5 — Figure S5: Limited influence of FOLFIRINOX components on IL‐18 induction in MIA PaCa‐2 cells. (A) MIA PaCa‐2 cells were treated with 5‐FU and/or levofolinate at the indicated concentrations for 48 h in low‐nutrient culture medium. Whole‐cell lysates were analyzed by western blotting with anti‐IL‐18 mAbs. β‐actin was used as a loading control. (B) MIA PaCa‐2 cells were treated with irinotecan (20 μg/mL) or oxaliplatin (2.5 μg/mL) alone or in combination with 5‐FU (25 μg/mL) for 48 h in low‐nutrient culture medium. Whole‐cell lysates were analyzed by western blotting with anti‐IL‐18 mAbs. β‐actin was used as a loading control. (C) MIA PaCa‐2 cells were treated with the four‐drug combination at the indicated concentrations for 48 h in low‐nutrient culture medium. Whole‐cell lysates were analyzed by western blotting with anti‐IL‐18 mAbs. β‐actin was used as a loading control. [file GTC-31-0-s001.pptx]

## Slide 1
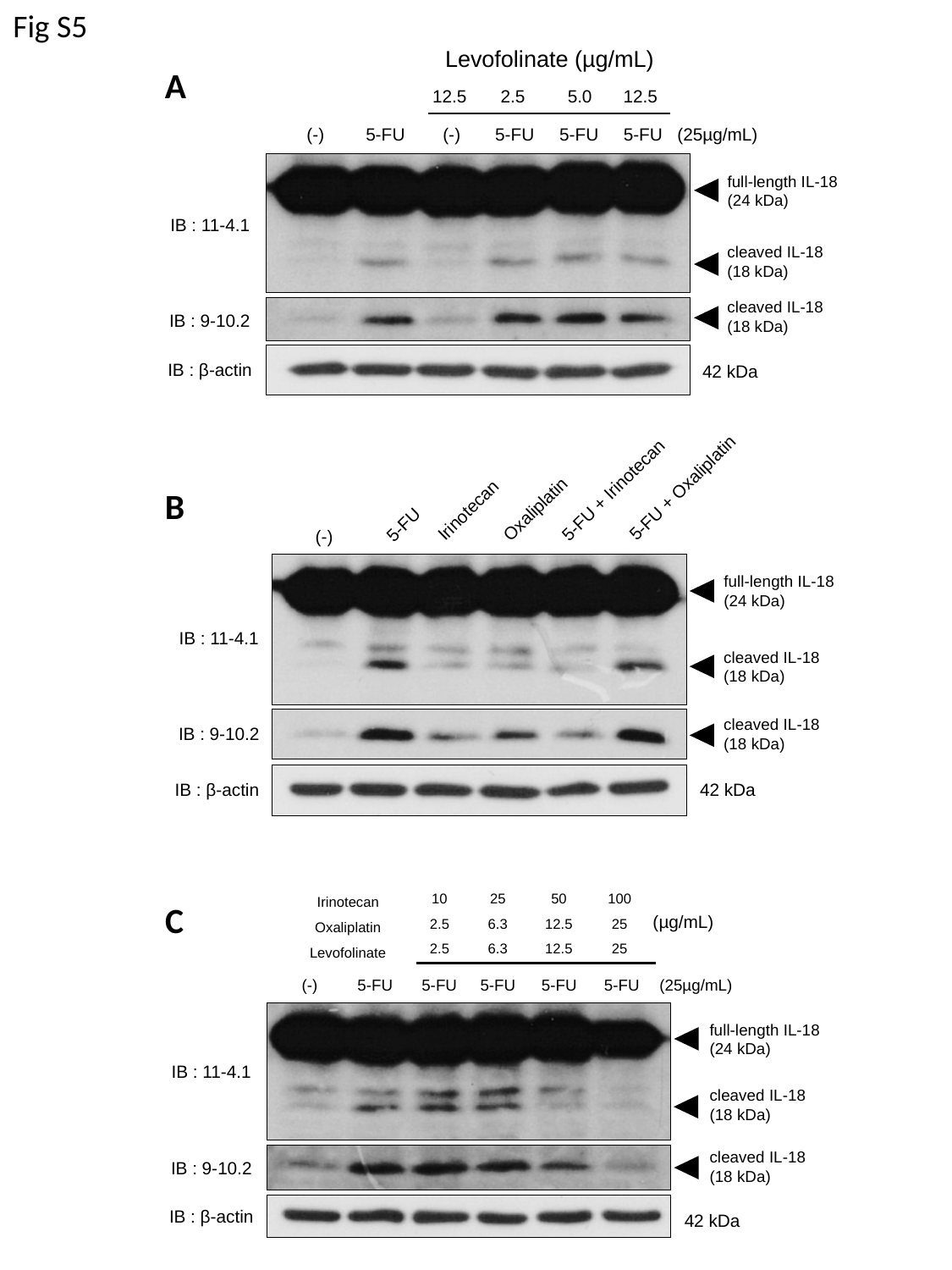

Fig S5
Levofolinate (µg/mL)
A
12.5
5.0
2.5
12.5
(-)
5-FU
(-)
5-FU
5-FU
5-FU
(25µg/mL)
full-length IL-18
(24 kDa)
IB : 11-4.1
cleaved IL-18
(18 kDa)
cleaved IL-18
(18 kDa)
IB : 9-10.2
IB : β-actin
42 kDa
5-FU + Oxaliplatin
5-FU + Irinotecan
B
Oxaliplatin
Irinotecan
5-FU
(-)
full-length IL-18
(24 kDa)
IB : 11-4.1
cleaved IL-18
(18 kDa)
cleaved IL-18
(18 kDa)
IB : 9-10.2
42 kDa
IB : β-actin
Irinotecan
Oxaliplatin
Levofolinate
10
25
50
100
C
(µg/mL)
2.5
6.3
12.5
25
2.5
6.3
12.5
25
(-)
5-FU
5-FU
5-FU
5-FU
5-FU
(25µg/mL)
full-length IL-18
(24 kDa)
IB : 11-4.1
cleaved IL-18
(18 kDa)
cleaved IL-18
(18 kDa)
IB : 9-10.2
IB : β-actin
42 kDa
